# Permanent Draft Genome Sequences of Three *Frankia* sp. Strains That Are Atypical, Noninfective, Ineffective Isolates

**DOI:** 10.1128/genomeA.00174-17

**Published:** 2017-04-13

**Authors:** Abdellatif Gueddou, Erik Swanson, Amir Ktari, Imen Nouioui, Karima Hezbri, Faten Ghodhbane-Gtari, Stephen Simpson, Krystalynne Morris, W. Kelley Thomas, Arnab Sen, Maher Gtari, Louis S. Tisa

**Affiliations:** a Université de Tunis El Manar, Tunis, Tunisia; b University of New Hampshire, Durham, New Hampshire, USA; c University of North Bengal, Siliguri, India

## Abstract

Here, we present draft genome sequences for three atypical *Frankia* strains (lineage 4) that were isolated from root nodules but are unable to reinfect actinorhizal plants. The genome sizes of *Frankia* sp. strains EUN1h, BMG5.36, and NRRL B16386 were 9.91, 11.20, and 9.43 Mbp, respectively.

## GENOME ANNOUNCEMENT

Endosymbiotic plant-bacterium associations are contributors to terrestrial biological nitrogen fixation and include actinorhizal symbiosis. This mutually beneficial symbiotic relationship between actinobacterial *Frankia* spp. and actinorhizal plants results in the formation of plant root nodule structure. This relationship allows proliferation of the plant through the bacterium, obtaining nutrients from the host plant in exchange for a source of fixed nitrogen that is assimilated by the host plant (1). Mutualistic infective *Frankia* strains are systematically classified based on their morphology, behavior in culture, and mode of infection within one of three major phylogenetic clusters (2). Another *Frankia* group isolated from actinorhizal nodules that are unable to undertake the nitrogen fixation process (Fix^-^) and/or reinfect their host plant causing nodulation (Nod^-^) are classified as “atypical *Frankia*” spp. and form a fourth phylogenetic cluster within the genus *Frankia*. The phenomena of how these atypical *Frankia* spp. enter inside nodule and the host metabolic cost of their presence as parasitic cheaters remain unclear (3). Although genomes for representatives for all four clusters have been sequenced (4), only two genomes are available for atypical *Frankia* spp. from cluster 4. The purpose of this study was to expand the number of genomes sequenced from cluster 4 to provide insight on these questions.

*Frankia* sp. strains EUN1h, BMG5.36, and NRRL B16386 were isolated from *Elaeagnus umbellata* (Tunisia), *Coriaria myrifolia* (Algeria), and *Morella californica* (United States; A. Gueddou, M. Gtari, M. Lechevalier, unpublished data), respectively. All three strains have failed to reinfect and nodulate their respective original host and any other actinorhizal host plant tested.

Sequencing of the draft genomes of *Frankia* sp. strains EUN1h, BMG5.36, and NRRL B16386 was performed at the Hubbard Center for Genome Studies (University of New Hampshire, Durham, NH) using Illumina technology techniques (5). A standard Illumina shotgun library was constructed and sequenced using the Illumina HiSeq 2500 platform with paired-end reads of 2 × 250 bp, which generated 2,121,668 to 15,077,492 reads (Table 1). The Illumina sequence data were trimmed by Trimmonatic version 0.32 (6) and assembled using SPAdes version 3.5 (7) and ALLPaths-LG version r52488 (8). Data on the final draft assemblies for *Frankia* sp. strains EUN1h, BMG5.36, and NRRL B16386 are presented in Table 1. The final assembled genomes for *Frankia* sp. strains EUN1h, BMG5.36, and NRRL B16386 contained total sequence lengths of 9,910,952, 11,203,906, and 9,435,764 bp, respectively, with an average G+C content of 71% (Table 1). The assembled *Frankia* sp. strains EUN1h, BMG5.36, and NRRL B16386 genomes were annotated via the NCBI Prokaryotic Genome Annotation Pipeline (PGAP) and resulted in 7,928, 8,952, and 7,562 candidate protein-coding genes, respectively. Bioinformatic analysis of these three genomes by use of the antiSMASH program (9, 10) revealed that these genomes again provided high numbers of secondary metabolic biosynthetic gene clusters (Table 1), similar to previous findings (4, 11), and including potential compounds, like frankiamicin (12).

**TABLE 1:** Genome statistics

*Frankia* strain	No. of reads	*N*_50_ contig size (kb)	Assembly size (Mb)	No. of contigs	Sequencing depth (×)	No. of CDSs^a^	G+C content (%)	No. of biosynthetic gene clusters^b^	Accession no.
BMG5.36	2,121,668	84.9	11.20	280	28.0	8,952	71.26	33	MBLO00000000
NRRL B-16386	10,384,450	117.5	9.43	174	161.4	7,562	71.93	27	MOMC00000000
EUN1h	15,077,492	194.6	9.91	129	305.1	7,928	71.83	30	MBLN00000000

a CDSs, coding sequences.

b Biosynthetic gene clusters for natural products were identified by the use of the antiSMASH software (9, 10).

### Accession number(s).

The draft genome sequences have been deposited in GenBank under the accession numbers in Table 1.
